# Morphometric and volumetric analysis of lacrimal glands in patients with thyroid eye disease

**DOI:** 10.1038/s41598-023-43083-0

**Published:** 2023-09-28

**Authors:** Ana Starčević, Zoran Radojičić, Aleksandra Djurić Stefanović, Aleksandar Trivić, Ivan Milić, Marina Milić, Dragan Matić, Jovana Andrejic, Vuk Djulejic, Igor Djoric

**Affiliations:** 1 Laboratory for Multimodal Neuroimaging, Institute of Anatomy, Medical faculty, University of Belgrade, Belgrade, Serbia; 2 Faculty of Organizational Sciences, University of Belgrade, Belgrade, Serbia; 3 Center for Radiology, University Clinical Center of Serbia, Medical faculty, University of Belgrade, Belgrade, Serbia; 4 Clinic for Otorhinolaryngology and Maxillofacial Surgery, University Clinical Center of Serbia, Medical faculty, University of Belgrade, Belgrade, Serbia; 5 Clinic for Neurosurgery, University Clinical Center of Serbia, Medical faculty, University of Belgrade, Belgrade, Serbia; 6 Clinic for Cardiology, University Clinical Centre of Serbia, Belgrade, Serbia; 7 Faculty of Medicine, University of Belgrade, Belgrade, Serbia; 8 Center for Radiology, Neurosurgery Clinic, University Clinical Center of Serbia, Medical faculty, University of Belgrade, Belgrade, Serbia

**Keywords:** Diseases, Endocrinology

## Abstract

Assessment of morphometric and volumetric changes in lacrimal glands in thyroid eye disease, its clinical manifestations in relation of disease progression. Retrospective volumetric analysis included both genders and was performed on total of 183 patients - 91 patients with diagnosed Grave’s disease and thyroid eye disease and 92 patients without Grave’s disease and thyroid eye disease who underwent multidetector computed tomography (MDCT) examination in routine daily work according to other medical indications. In the group of females, there was statistical significance between patients with thyroid eye disease and controls who were smoking and had body weight gain. We found statistical significance in volumetric enlargements for both orbits in both genders for the patients group when compared to controls. There was also statistical significance in morphometric characteristics for the lacrimal gland diameters measured. Determination planimetric morphometric parameters of importance were coronary height of lacrimal gland of the right eye, coronary height of lacrimal gland of the left eye and coronary width of lacrimal gland of the left eye for the group of males. In a group of females the established determination parameters of importance were the coronary height of lacrimal gland of the left eye, the axial width of lacrimal gland of the left eye, volume of lacrimal gland of the right eye and the volume of lacrimal gland of the left eye. When we compared the displaced lacrimal gland coming forward (proptosis) in time progressing disease between group of patients and controls, we also found statistical significant connection. Evaluation of lacrimal gland volumetric and morphometric data may increase validity of defining this anatomical substrate and its morphology disruption as liable tool for thyroid eye disease progression follow up and treatment planning and outcome.

## Introduction

The lacrimal glands are paired amygdaloid glands located in the zygomatic process of frontal bone and specifically susceptible to autoimmune diseases such as Grave’s disease. Thyroid eye disease (TED), also known as Graves’ orbitopathy (GO) presents an autoimmune-driven ophthalmic clinical manifestation in Grave’s disease, and occurs primarily in patients with hyperthyroidism, but can also occur in patients with hypothyroidism and even euthyroidism. It can occur concurrently with endocrine abnormalities or may precede or follow them. Thyroid eye disease can induce periorbital oedema, erythema, proptosis, eyelid retraction, restrictive strabismus leading to diplopia, chemosis, and, in severe cases, increased intraocular pressure, exposure keratopathy, and even optic nerve compression. These symptoms might have serious physical and psychological consequences and impact the quality of life in many senses^1^. Thyroid eye disease may also have an impact on central nervous system function and cerebral cortical thickness inducing structural brain changes^2^. Displaced lacrimal gland coming forward and posterior herniation of the orbital fat are associated with optic neuropathy with a sensitivity of 94% and specificity of 91%^3^. Cigarette smoking is presumed to be the strongest risk factor for developing Grave’s orbitopathy^4^. Increased body weight and body mass index present also one of the risks for Grave’s orbitopathy and its complications^5^.

Currently, the disease is diagnosed using a combination of clinical signs and symptoms, laboratory tests, and imaging studies. MDCT is widely available, relatively inexpensive and highly reproducible testing methods of orbit at GO^6^. The progress assessment of TED and GO is determined by the Clinical Activity Score (CAS) by the presence or absence of seven inflammation signs or symptoms such as spontaneous retrobulbar pain, pain during movement of the eyes, redness of the eyelids, conjunctival redness, swelling of the eyelids, island lacrimalcaruncular lesions and conjunctival edema. CAS equal to 3 or greater suggests active GO^7–9^. Also, NOSPECS classification (No signs and symptoms, Only signs Soft tissue involvement, Proptosis, Extraocular muscle involvement, Corneal involvement and Sight loss), is used, which represents an ophthalmic mnemonic tool but with a lower use value^10,11^.

Computed tomography (CT) is a common imaging modality used in clinical practice for the diagnosis, treatment evaluation, and postoperative follow-up of thyroid eye disease. The use of Multi-detector row computed tomography (MDCT) in the diagnosis of GO enabled the production of large quantities of information in a relatively short time of the examination, but also a significant reduction of artifacts and errors in the measurement and evaluation of the orbital contents. Also, imaging software can reformat images in three planes (transaxial, coronary and sagittal) as well as high-resolution display images of orbital structures^12^. The quantitative relationship between the morphometric changes in lacrimal gland in patients with GO using planimetric and volumetric, morphometric analysis of orbital soft tissue presents no routine protocol so far^13–16^.

Our aim was to analyse and evaluate morphometric and volumetric characteristics of lacrimal glands in patients with thyroid eye disease by MDCT.

## Materials and methods

Retrospective volumetric analysis was done on 91 patients (19 males and 72 females aged from 18 to 80 with mean age 49.49 ± 12.02) diagnosed with Grave’s disease and TED. The patients included in this investigation were tested to TSH receptor antibodies by endocrinologist before MDCT examination. The study group patients included in this investigation had Graves’ disease coexisting with hyperthyroidism, hypothyroidism or euthyroidism by an endocrinologist. All study group patients were diagnosed de novo and there were no pharmacological treatment before our MDCT examination. Grave’s ophthalmopathy (GO) was considered to be present if eyelid retraction occurred in association with thyroid dysfunction, exophthalmos, extraocular muscle involvement or optic nerve disruption according to ophthalmologist as previously described^17^. We evaluated diagnostical volumetric and morphometrical radiological parameters but not therapeutical status of these patients.

The control group consisted of 92 patients (44 men and 48 women aged from 18 to 80 with mean age 52.15 ± 14.44) without Grave’s disease and TED. The control group of patients was formed based on the clinical picture and history. They were not TSH measured, because neither one patient included in control group did not have an endocrine disease or suspected endocrine disease, but was included by the method of random selection for the control group (underwent multidetector computed tomography examination in routine daily work according to other medical indications-sinusitis, headaches, ptosis, intracranial aneurysm, facial nerve paralysis, cranial nerve palsy). The inclusive parameter for entering the control group of patients was that they do not have thyroid disease, tumor or inflammation related to endocrine disease. The TSH receptor antibodies were not tested to control patients group nor was the TSH level determined.

All patients underwent MDCT examination of region of interest-orbit. MDCT examination of orbit was performed with 64-slice GE Healthcare LightSpeed VCT 64-slice CT Scanner scanner in routine 0.625 mm slice thickness endocranium protocol scanners. All examinations were performed MDCT natively first, followed by the intravenous administration of iodine contrast (Ultravist 300 mg/mL) in an amount of 50 mL, delayed 40 seconds.

Before the MDCT examination, an interview was conducted with all patients. Patients who were allergic to iodine contrast agent, had previous reaction to any contrast agent and/or antibiotic, had an increased risk of developing heart rhythm disorders, had previous had epileptic seizures, had impaired kidney and liver function in a short period before the MDCT examination, underwent MDCT of another region for another indication, which involved the use of an iodine contrast medium or were female patients pregnant or breastfeeding were not examined by MDCT in our investigation. After each of our MDCT examinations (study and control group), the patients were under 24-hour monitoring for any form of allergic reaction. No form of allergy to iodine contrast medium or any type of adverse reaction has been reported. In our study, we used Ultravist 300, which contains the active substance iopromide, which belongs to the group of nephrotropic low-osmolar X-ray contrast agents. Although the diagnostic recommendations for dosing Ultravist 300 iodinated contrast medium are 1-2 mL/kg of body weight, we conducted to all our patients a dose of only 50 mL, which in our clinical practice proved to be more than sufficient for examination and precise radiological computed tomography of orbit diagnostics. And in this way, we prevented and reduced the risk of "iodine-based contrast media-induced thyroid dysfunction". It was shown that there is connection between immediate exposure to elevated iodide levels that can induce disruption of thyroid function and incident hyperthyroidism and hypothyroidism occurrence^18–21^. The Institutional Review Board of the University Clinical Centre of Serbia and Ethical Committee University Clinical Centre Medical faculty University of Belgrade approved this study (under number 29/IV-13).All participants need/requirement for informed consent was waived by ethical committee of the University Clinical Centre of Serbia Medical faculty University of Belgrade. All methods were performed in accordance with the relevant guidelines and regulations and informant consent was provided.

### Image analysis

**Figure 1:**
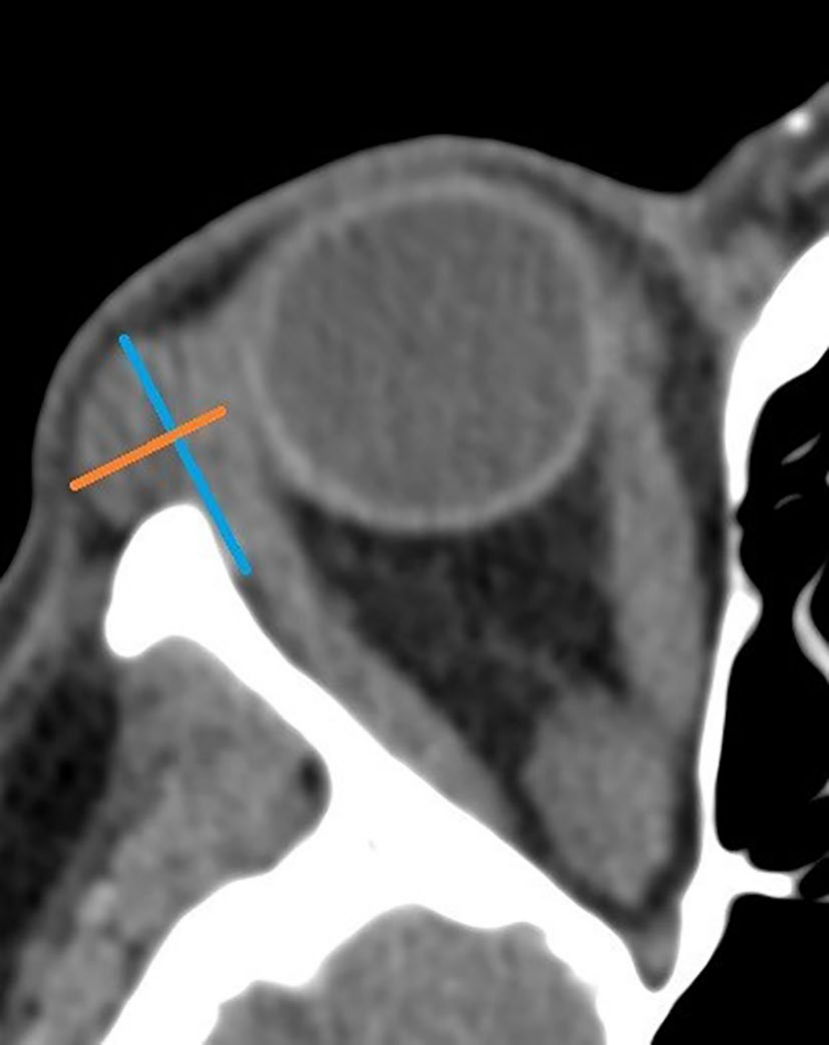
Schematic view of the value of the axial length (marked in blue) and the axial width (marked in orange).

**Figure 2:**
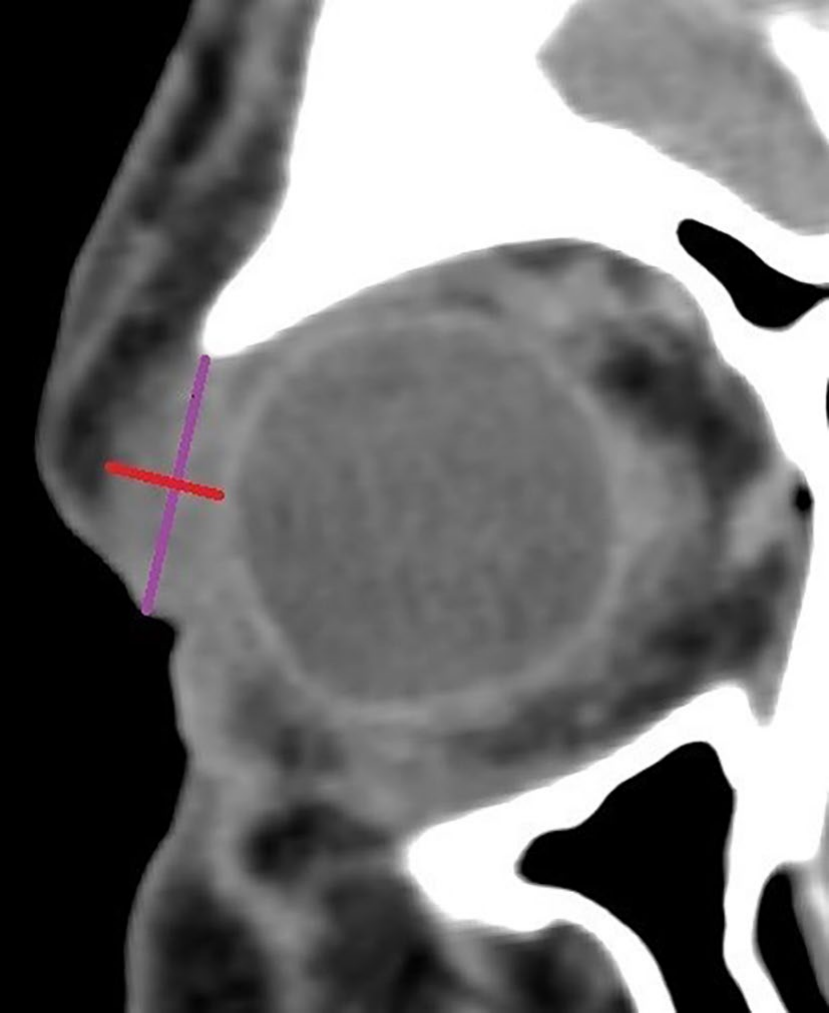
Schematic view of the value of coronary length, and high (marked in purple) and coronary width (marked in red).

The volume rendering images of the lacrimal glands were obtained separately for the right (OD) and the left orbit (OS). Two neuroradiologists and one neuroscientist independently inspected MDCT scans. After interobserver evaluation agreement was agreed, the neuroscientist evaluated the remaining measurements. Volume rendering techniques (VRT) with MDCT allow excellent 3D reconstructions that help radiologists to visualize and analyse morphological anomalies in anatomical substrates as well as to evaluate their relation to other anatomical substrates and make specific treatment plans^19,21^. After using a tool for reorientation of DICOM images both axial and coronal images and volume dimensions of lacrimal glands were estimated. The MDCT parameters that were analyzed are lacrimal gland diameters (axial length, axial width, coronary height, and width) and volumes of the lacrimal gland (Figs. 1 and 2). Important parameter was if dislocated lacrimal gland coming forward, exophthalmos, proptosis, existed. In the examined axial image, the length was calculated from the most anterior point to the most posterior point of the gland and the width was calculated from the most lateral to the most medial point at the widest point perpendicular to the length at the same selected image slice. The examined anatomical substrate was delineated with a free hand technique by semiautomatic delineation tool in all consecutive images at the examined lacrimal gland. The volume of region of interest was calculated by the software 3D Slicer package. The results of 3D CT volume rendering images were compared with the axial images in order to calculate and test diagnostic sensitivity, specificity and accuracy.

### Statistical analysis

Data analysis was performed using the method of descriptive analysis and exploratory analysis. Student *t* test was used to compare two independent groups. Pearson Chi-Square, odds ratio (OD) and relative risk (RR) were used to compare categorical parameters. Raw under the curve (ROC) was used for the level of sensitivity and specificity. The area under the curve (AUC) was used for testing the sensitivity of parameters. The cutoff point was defined as the positive/negative ratio (P/N ratio). Pearson correlation coefficient and coefficient of determination were used as a measure of linear association. Spearman correlation coefficient was used as a measure of association between scores. Significance was accepted at p<0.05. The power of tests was on level 80% or more. Data are presented as mean ± SD or median and range. Data analysis was done with SPSS v20.0 statistical software.

### Ethical approval

The Institutional Review Board of the University Clinical Centre of Serbia and Ethical Committee University Clinical Centre Medical faculty University of Belgrade approved this study (under number 29/IV-13). All participants need/requirement for informed consent was waived by ethical committee with full name. All methods were performed in accordance with the relevant guidelines and regulations and informant consent was provided.

## Results

Parameters included in this investigations were demographic, anthropometric (morphometric) and clinical (age, gender, smoking age experience, the number of cigarettes, body weight, height, body mass index (BMI), duration of disorders of thyroid function and duration of ocular signs).

Demographic characteristics are shown in Table 1. The results showed no statistical significance between patients with TED and controls in the group of males in demographic parameters. In the group of females, there was statistical significance between patients with TED and controls who were smoking. In the group of females there was a statistically significant difference between patients and controls expressed in a greater number of smokers in specific affected group p=0.000 (38 of 72; 53% of those affected in comparison to the control 5 of 48; 10%). There was statistical significance in the group of females related to weight for those with greater weight p=0.000 (72.72 +/– 7.14 kg in comparison to the controls 64.13 +/– 10.85 kg) and parameter BMI in which the group of patients had a higher BMI p=0.006 (27.12 +/– 5.2 kg/m^2^ when compared to controls 24.6+/– 4.1 kg/m^2^). Other parameters showed no statistically significant differences.

**Table 1:** Demographic, antropometric and clinical parameters of the patients and control group.

Parameters	Group	Gender							
		Male				Female			
		N	Mean	SD	p	N	Mean	SD	p
Age	Patients	19	50.421	12.438	0.481	72	49.250	11.979	0.517
	Control	44	53.295	15.662		48	51.104	17.141	
Smoking	Patients	19	Yes/no	14/6	0.662	72	Yes/no	38/34	0.000
	Control	44	Yes/no	30/15		48	Yes/no	5/43	
Age of smoking	Patients	14	25.500	11.713	0.247	37	23.865	10.253	0.269
	Control	30	30.600	14.122		5	29.200	7.294	
No of cigarettes per day	Patients	14	31.429	12.924	0.623	38	21.579	10.274	0.934
	Control	30	29.667	9.994		5	22.000	13.038	
Height	Patients	19	175.263	5.476	0.755	72	163.889	6.200	0.075
	Control	44	175.977	12.573		48	161.604	7.687	
Weight	Patients	19	87.632	17.982	0.711	72	72.722	14.070	0.000
	Control	44	86.023	14.707		48	64.125	10.848	
BMI	Patients	19	28.031	4.651	0.761	72	27.120	5.195	0.006
	Control	44	27.702	3.556		48	24.596	4.123	
Duration of thyroid disease	Patients	19	48.053	51.929	–	72	49.597	59.613	–
	Control	0				0			
Duration of eyes disorder	Patients	19	30.842	35.428	–	72	27.972	45.493	–

The morphometric and volumetric data analysis was conducted and obtained separately for the right and left orbit (Table 2, Figs. 1, 2).

**Table 2:** Morphometric and planimetric differences between left and right orbits in diameters and volumes.

Parameters	Male					Female				
	Group	N	Mean	SD	P	Group	N	Mean	SD	p
Diameter axial length OD	Pat	19	17.784	3.513	0.001	Pat	72	16.931	2.988	0.000
	Con	44	14.43	2.64		Con	48	14.921	2.688	
Diameter axial width OD	Pat	19	5.789	1.952	0.008	Pat	72	5.572	1.25	0.000
	Con	44	4.423	0.974		Con	48	4.602	1.223	
Coronary diameter axial length OD	Pat	19	17.784	2.862	0.000	Pat	72	16.863	3.11	0.000
	Con	44	14.593	2.33		Con	48	14.921	2.124	
Coronary diameter axial width OD	Pat	19	4.947	1.486	0.011	Pat	72	5.063	1.152	0.000
	Con	44	4.077	1.082		Con	48	4.127	0.914	
Volume OD	Pat	19	0.763	0.451	0.002	Pat	72	0.735	0.25	0.000
	Con	44	0.499	0.196		Con	48	0.521	0.25	
Diameter axial length OS	Pat	19	17.058	3.456	0.008	Pat	72	16.403	2.927	0.002
	Con	44	14.545	2.425		Con	48	14.644	2.893	
Diameter axial width OS	Pat	19	5.758	2.14	0.018	Pat	72	5.5	1.309	0.000
	Con	44	4.439	1.009		Con	48	4.531	1.165	
Coronary diameter axial length OS	Pat	19	16.647	3.346	0.025	Pat	72	17.388	2.843	0.000
	Con	44	14.98	2.292		Con	48	15.142	2.291	
Coronary diameter axial width OS	Pat	19	5	1.047	0.000	Pat	72	4.918	1.275	0.001
	Con	44	3.977	0.974		Con	48	4.135	1.038	
Volume OS	Pat	19	0.753	0.49	0.039	Pat	72	0.719	0.24	0.000
	Con	44	0.497	0.181		Con	48	0.554	0.245	

*OD* right orbit, *OS* lest orbit.

There was statistical difference p = 0.002 (0.763 +/– 0.451 cm^3^ vs. 0.499 +/– 0.196 cm^3^) in a group of male patients and controls when volumes of the right orbits were compared as well as for the left orbits at the level of p = 0.039 (0.753 +/– 0.490 cm^3^ vs 0.497 +/– 0.181 cm^3^).

We also found statistical significance in a group of female patients and controls when volumes of the right orbits were compared p = 0.000 (0.735 +/– 0.250 cm3 vs. 0.521 +/– 0.250 cm3), and for the left orbits statistically significant difference was at the level of p = 0.000 (0.719 +/– 0.240 cm^3^ vs 0.554 +/– 0.245 cm^3^).

**Figure 3:**
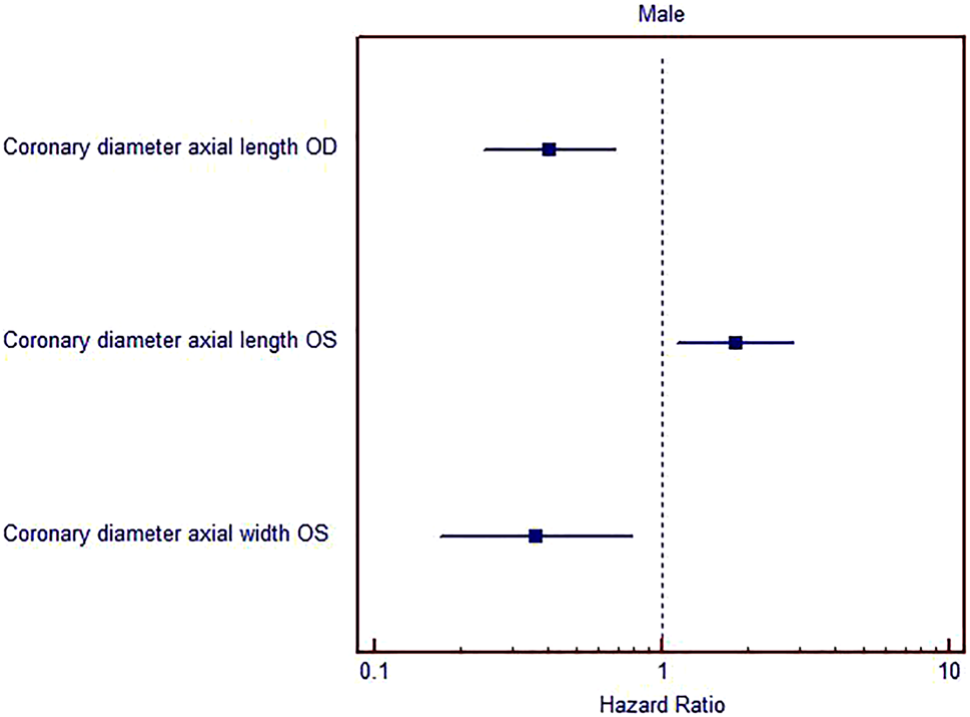
Multivariate binomial logistic regression for estimation of the most important parameters for hazard ratio (Male). *OD* right orbit, *OS* left orbit.

To determine the parameters of importance for the expression level of the disease, the multivariate binomial logistic regression was applied in a group of males. Determination planimetric morphometric parameters of importance were coronary height of lacrimal gland of the right eye (p = 0.001), coronary height of lacrimal gland of the left eye (p = 0.013) and coronary width of lacrimal gland of the left eye (p = 0.011) (Fig. 3).

**Figure 4:**
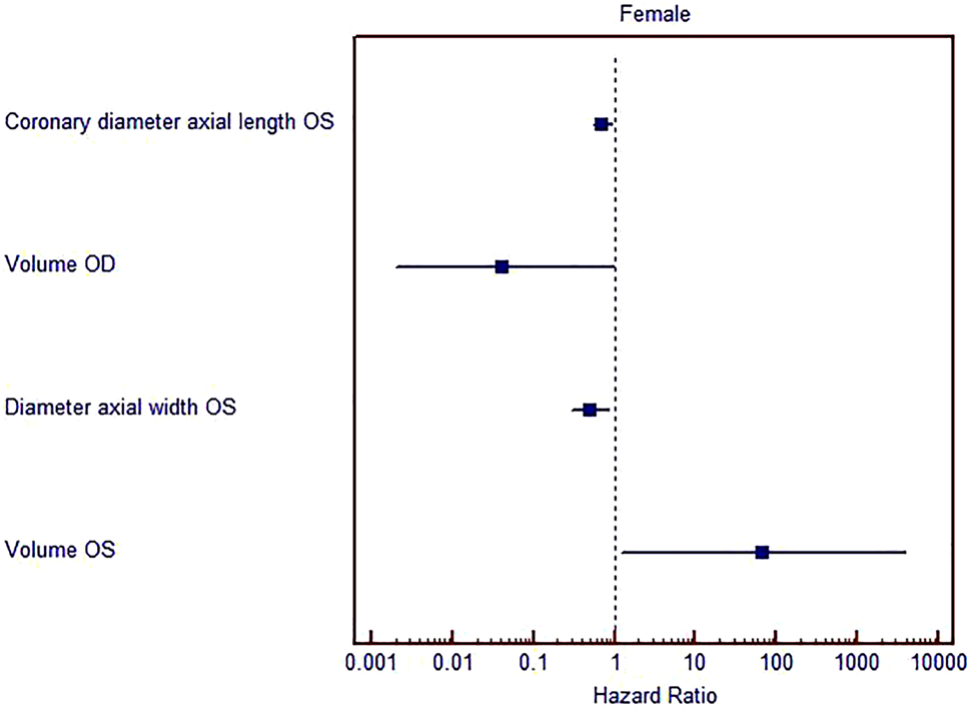
Multivariate binomial logistic regression for estimation of the most important parameters for hazard ratio (Female).

Multivariate binomial logistic regression showed established determination parameters of importance, coronary height of lacrimal gland of the left eye (p = 0.007), the axial width of lacrimal gland of the left eye (p = 0.008), volume of lacrimal gland of the right eye (p = 0.048) and the volume of lacrimal gland of the left eye (p = 0.040) in a group of female patients (Table 3, Fig. 4).

**Table 3:** Multivariate binomial logistic regression, method stepwise forward conditional for estimation of the most important parameters.

Gender	Parameters	OR	p	95% CI lower	95% CI upper
Male	Coronary diameter axial length OD	0.405	0.001	0.239	0.689
	Coronary diameter axial length OS	1.804	0.013	1.133	2.872
	Coronary diameter axial width OS	0.365	0.011	0.168	0.790
	Constant	42939.845	0.001		
Female	Coronary diameter axial length OS	0.712	0.007	0.558	0.910
	Volume OS	0.042	0.048	0.002	0.969
	Diameter axial width OS	0.5	0.008	0.300	0.834
	Volume OS	68.678	0.040	1.222	3860.159
	Constant	2553.667	0.001		

*OS* left orbit, *OD* right orbit.

**Figure 5:**
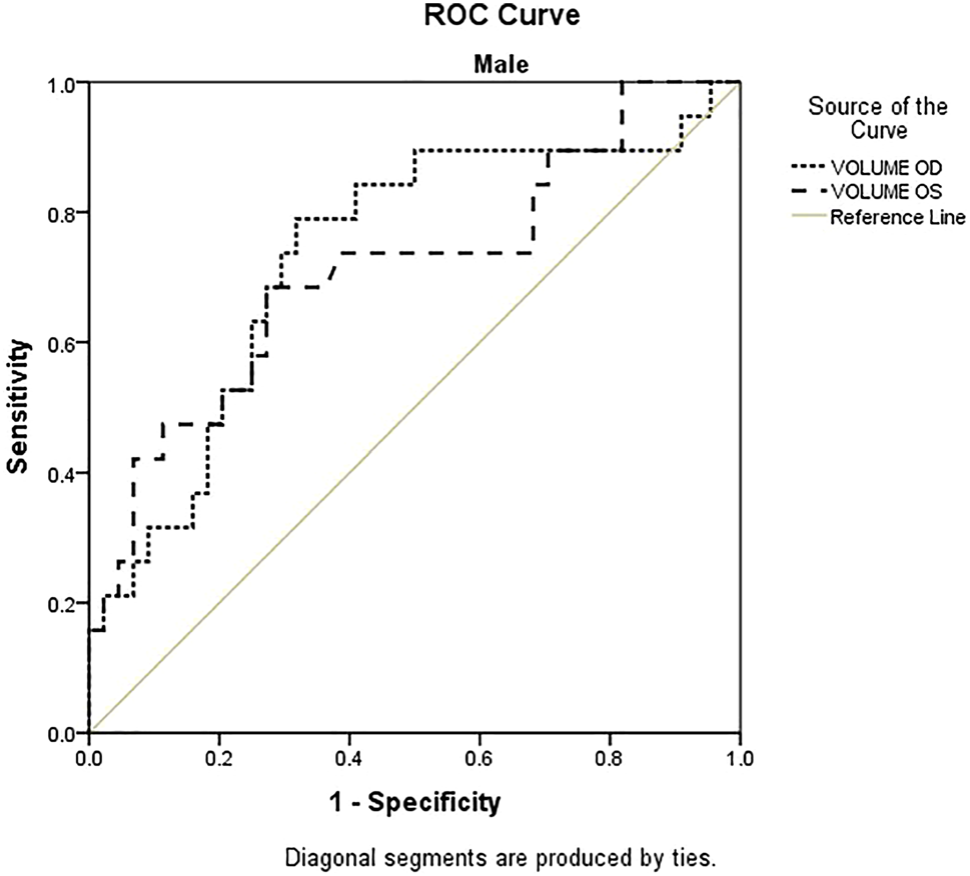
ROC curve for the volumes of the right (OD) and left orbits (OS) in men.

**Figure 6:**
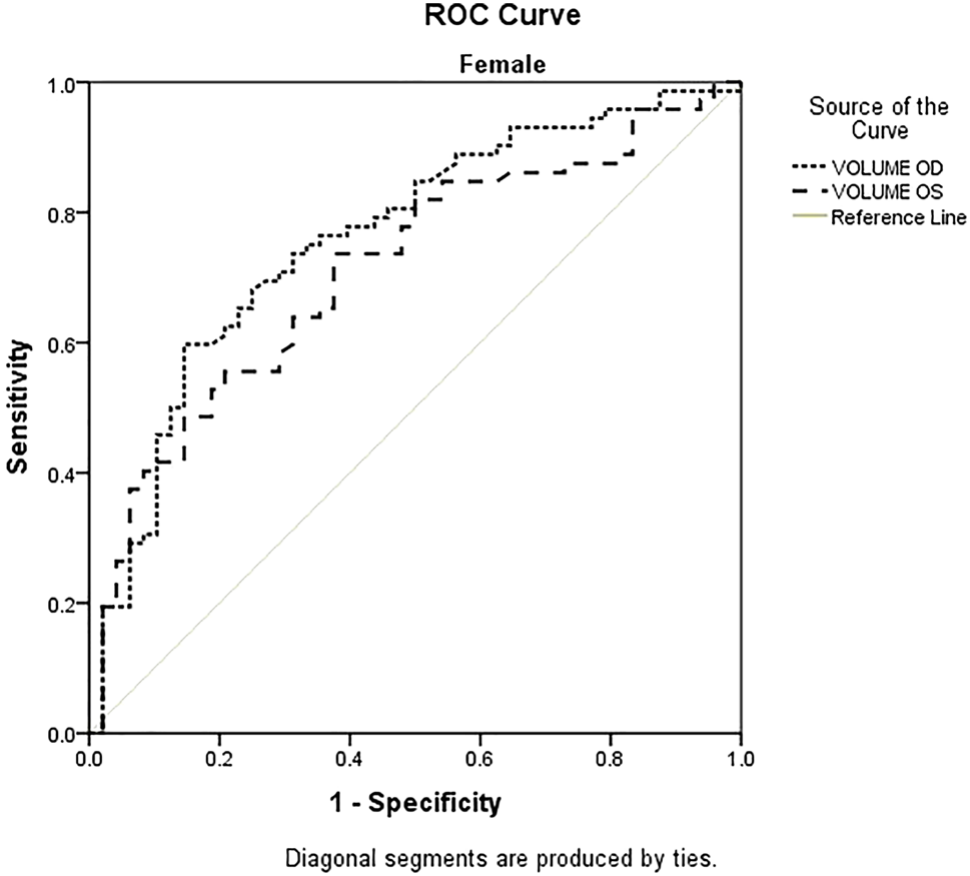
ROC curves for the volumes of the right (OD) and left (OS) orbits in women. *OD* right orbit, *OS* left orbit.

The cutoff values for specific different parameters are showed with a ROC curve for both genders, female and male groups (Figs. 5 and 6).

**Figure 7:**
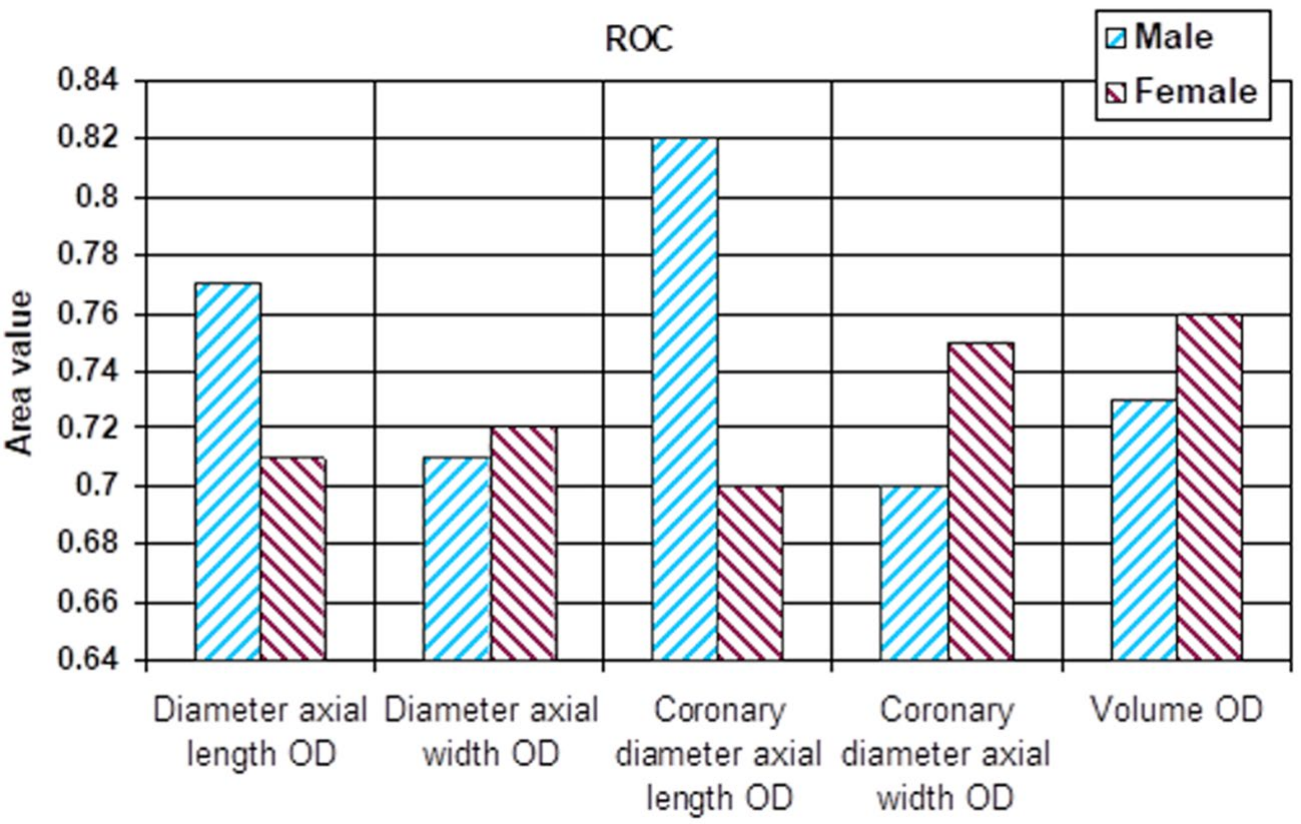
ROC comparison between male and female patients (right orbit-OD).

**Figure 8:**
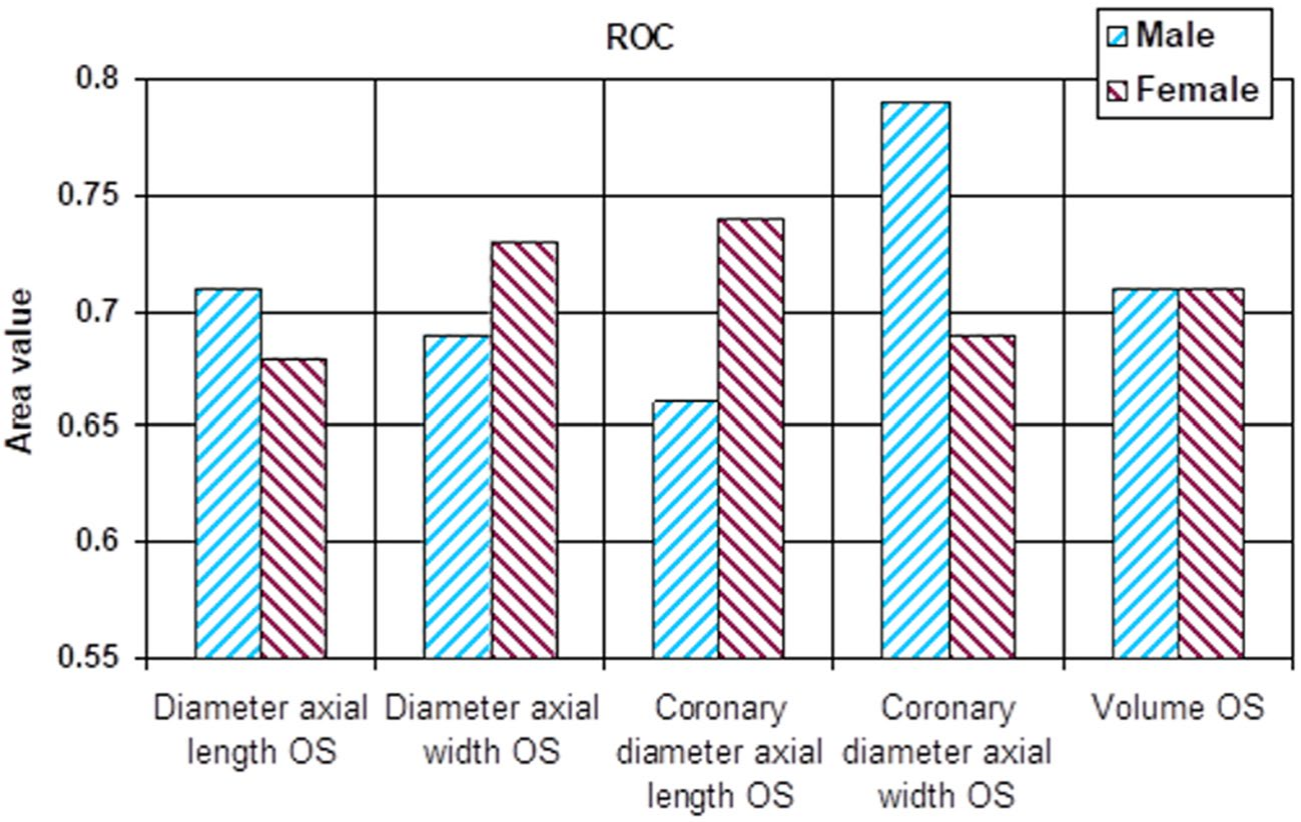
ROC comparison between male and female patients (left orbit-OS).

All monitored parameters were statistically significant for determining the sensitivity and specificity (Table 4, Figs. 7 and 8).

**Table 4:** ROC curve with cutoff point based on P/N ratio.

Parameters	Male					Female				
	Area	p	95% confidence interval		Cut off	Area	p	95% confidence interval		Cut off
			Lower bound	Upper bound	Value for P/N ratio			Lower bound	Upper bound	Value for P/N ratio
Diameter axial length OD	0.77	0.00	0.63	0.89	15.40	0.71	0.00	0.61	0.80	15.70
Diameter axial width OD	0.71	0.01	0.55	0.87	4.80	0.72	0.00	0.62	0.80	5.00
Coronary diameter axial length OD	0.82	0.00	0.70	0.93	15.90	0.70	0.00	0.60	0.79	15.50
Coronary diameter axial width OD	0.70	0.01	0.55	0.83	4.20	0.75	0.00	0.66	0.84	4.50
Volume OD	0.73	0.00	0.59	0.87	0.59	0.76	0.00	0.67	0.84	0.58
Diameter axial length OS	0.71	0.01	0.56	0.85	15.20	0.68	0.00	0.58	0.77	15.50
Diameter axial width OS	0.69	0.02	0.54	0.84	4.80	0.73	0.00	0.63	0.82	4.90
Coronary diameter axial length OS	0.66	0.05	0.51	0.81	15.30	0.74	0.00	0.64	0.82	15.90
Coronary diameter axial width OS	0.79	0.00	0.67	0.90	4.40	0.69	0.00	0.59	0.80	4.30
Volume OS	0.71	0.01	0.56	0.86	0.56	0.71	0.00	0.62	0.80	0.61

When we compared the displaced lacrimal gland coming forward between group of patients and controls, we found statistical significant connection (p = 0.000). The sensitivity of displaced right lacrimal glands coming forward (OD) is 73.6%, while the specificity of 77.2%. Odds ratio was OR = 9.438 (4.810–18.521), and the relative risk of displacement, proptosis is RR = 3.226 (2.172–4.790) (Table 5).

**Table 5:** Displacement of lacrimal glands way forward (right orbit-OD).

			Displacement OD		Total
			Yes	No	
GO	Yes	Count	67	24	91
		% within	73.6%	26.4%	100.0%
	No	Count	21	71	92
		% within	22.8%	77.2%	100.0%
Total		Count	88	95	183
		% within	48.1%	51.9%	100.0%

When we compared the displaced lacrimal gland coming forward between group of patients and controls, we found statistical significant connection (p = 0.000). The sensitivity of displaced left lacrimal glands coming forward (OS) is 70.3%, while the specificity 78.3%. The odds ratio was OR = 8.533 (4.370–16.662), and the relative risk of displacement amounts to RR 3.235 (2.147–4.875) (Table 6).

**Table 6:** Displacement of lacrimal glands way forward (left orbit-OS).

			Displacement OS		Total
			Yes	No	
GO	Yes	Count	64	27	91
		% within	70.3%	29.7%	100.0%
	No	Count	20	72	92
		% within	21.7%	78.3%	100.0%
Total		Count	84	99	183
		% within	45.9%	54.1%	100.0%

## Discussion

Thyroid eye disease presents the most common extrathyroidal manifestation of Graves’ disease, autoimmune inflammatory disorder affecting the orbital tissues. Thyroid diseases occur generally more commonly in women than in men^19,21^. TED is not common, it occurs in a certain percentage of patients with Graves’ disease predominantly women. Our results also showed that persistence of left lacrimal gland displaced forward according to the diameters as more specific in women compared to males. In one of similar studies conducted, men demonstrated more asymmetric dynamics of disease like proptosis and overall asymmetry than women, while hyperthyroid females demonstrated more symmetry than euthyroid and hypothyroid males and females but in the same study, NO SPECS severity score was unaffected by gender, thyroid status, or symmetry. Greater sensitivity and specificity was observed in morphometric characteristics in left lacrimal glands in both gender showing potential in disease progression in our study. Previous investigations showed that the magnitude of lacrimal gland structural changes varied by gender emphasizing that the sexual dimorphism was most visible in men at all ages except pre-weaning and that male lacrimal tissue had considerably higher acinar area than females, which make important anatomical characteristics^22^. Previous animal studies has showed gender-related differences exist in lacrimal glands and that are correlated with morphological parameters of the lacrimal gland, as well as the volume, protein and IgG content of tears, measured in male and female infant, pre-weanling, pubertal, adult and old rats. In the same study, age-related variations occurred in the weight and morphological appearance of the lacrimal gland with the magnitude of structural changes dependent upon gender^23^. Clinical correlations with statistical significance conjoint with disrupted morphometric and volumetric data may pinpoint to disease progression in time. Our investigation showed statistical significance between patients with TED and controls who were smoking, number of cigarettes and greater body weight in the group of females. These findings are in the consistency with previous investigations that provided strong evidence for a causal association between smoking and development of TED. Smokers were also more likely to experience disease progression or poorer outcome of treatment. However, there were studies proving non correlation between smoking and TED, stating that there are no direct significant values correlating smoking habit to thyroid volume or function^24^. Results from a study conducted with smokers with Graves' disease showed that they were more likely to experience rapid eye deterioration, including the development of double vision, the constriction of eye movement, and irreversible optic nerve damage^25^. On the other side, no statistically significant differences were observed in the body weight changes of hypothyroid and hyperthyroid patients, neither at diagnosis, nor following normalization of hormone levels after treatment in similar study^26^. Thyroid diseases generally occur more commonly in women than men^27^. Potential cause refers to the fact that thyroid dysfunction is associated with changes in body weight and composition, body temperature and total and resting energy expenditure independently of physical activity. Moreover, there is a fact that weight gain often develops after treatment of thyroid dysfunction. Obesity and thyroid dysfunction are common diseases, and consequently clinicians should be particularly alert to the possibility of thyroid dysfunction in obese patients and therefore correlation with TED^28^. Treatment of hyperthyroidism commonly results in weight gain, but the extent of weight gain is not well known, some of them may refer to psychological issues and psychiatric disorders. Patients may regain the weight they had lost or may overshoot and become obese^29^. The alteration of hormonal status may impact lacrimal glands morphology and function as well. Testosterone allows normal support and increased tissue proliferation of lacrimal glands, therefore chronic exposure to androgen receptor antagonists results in degenerative changes in lacrimal glands and volume reduction. According to previous investigations, estrogens’ role in anatomy of lacrimal glands is very debatable. While some researchers claim minimal invasiveness of estrogen in morphology of lacrimal glands others state adverse influence on the lacrimal gland, such as inducing glandular regression, acinar cell disruption, and necrosis^30,31^. Characteristic ophthalmic signs of TED that sometimes precede laboratory analysis include exophthalmos, eyelid retraction, eyelid oedema, restrictive extraocular myopathy, and optic neuropathy associated with thyroid dysfunction. Lacrimal gland presents anatomical substrate specifically affected in its volume and shape with emphasize on unilateral lacrimal gland enlargement which may present the predominate early clinical sign^32^. Our results showed both orbits alterations in volume and morphometric, planimetric characteristics in both genders. Also, when we compared the displaced lacrimal gland coming forward-proptosis between group of patients and controls, we found statistical significant connection in cases of both left and right lacrimal glands with slight greater statistical relevance in the case of left lacrimal gland. Lacrimal gland size and imaging characteristics are important since lacrimal gland may be defined as very significant anatomical, morphometric substrate and possible prognostic determinant in many specific diseases. Previous studies in the literature reported different sizes of lacrimal gland between different ethnicities. The first study which refers to lacrimal gland dimensions in healthy participant with CT showed significant difference only in mean coronal length between the right and left orbits, other dimensions were similar between two sides, compared to our results which showed significant results when it comes to the axial width of left lacrimal gland and coronal hights of both left and right lacrimal glands^28,33^.

## Conclusion

Our investigation showed persistence of left lacrimal gland displaced forward according to the measured diameters as more specific in women with TED compared to males and its correlation with clinical data like smoking and obesity also more specific in females. MDCT structural imaging of lacrimal glands in patients with TED present a powerful diagnostic tool in clinical practice to diagnose and monitor the severity or progression of the disease along with other tests and examinations to help early diagnostics, treatment and managing TED. Volumetric and planimetric, morphometric lacrimal data correlate with statistical significance of clinical data making it possible morphological substrate for determination of Grave’s disease progression and dynamics and therefore MDCT routine scanning in conditions of medical indication presents a good pathway of observing and treatment modification of Grave’s disease. Future research with multiparametric CT imaging will improve the results by incorporating advanced post-processing techniques such as machine learning and texture analysis. A larger-scale multicentric study is needed to determine established dimensions of LGs that are representative of TED manifestation in both genders.

## Data Availability

The datasets used and/or analysed during the current study available from the corresponding author on reasonable request.
